# Accuracy and Reliability of CBCT Compared to Clinical Probing in Detection of Trifurcation Defects: An In Vivo Study

**DOI:** 10.1155/2022/5805776

**Published:** 2022-04-14

**Authors:** Abdulaziz Mohammad Alsakr, Adriana G. Creanga, Abdullah Saad Alqahtani, Khalid Gufran

**Affiliations:** ^1^Department of Preventive Dental Sciences, College of Dentistry, Prince Sattam Bin Abdulaziz University Alkharj, Saudi Arabia; ^2^Department of Diagnostic Sciences, Rutgers School of Dental Medicine, Newark, NJ, USA

## Abstract

**Background:**

Furcation defects are areas of pathological bone resorption in multirooted teeth. The aim of the study was to compare the measurements of trifurcation bone loss, measured using CBCT, versus clinical measurements in order to evaluate the efficacy of CBCT as an adjunctive diagnostic tool. *Material and Methods*. The included patients had both CBCT scans for maxillary molars and completed periodontal charts. Clinical examination consisted of probing and detection of vertical and horizontal furcation defects. These were measured and recorded. CBCT measurements were then evaluated using the linear measuring tool in Carestream imaging software (Carestream, Rochester, USA) and iCAT (Imaging Sciences, Hatfield, USA). These measurements of the CBCT images were then documented and compared to clinical findings. The two examiners were blinded to each other's measurements.

**Results:**

The most common tooth with a detected furcation defect was tooth #2 (31.7%), followed by tooth #15 (26.8%) and #3 (21.9%). The least common tooth with a detected furcation defect was #14 (19.5%). The mean values of buccal furcation for clinical and CBCT measurements were 3.01 mm and 2.6 mm, respectively. The measurements of mesial furcation were 2.5 mm and 2.2 mm for CBCT. The distal measurement of clinical examination was 2.7 mm and for CBCT was 2.44 mm.

**Conclusion:**

CBCT can be used as an adjunct to clinical furcation measurements and adds useful diagnostic information to assess trifurcation defects. In addition, CBCT limited field of view (FOV) can provide relatively high-resolution images at a reduced dose that is comparable to two-dimensional imaging.

## 1. Introduction

Plaque accumulation and the inflammatory process are, in the long term, the main etiological factors for furcation defects. Furcation defects are areas of pathological bone resorption in multirooted teeth, where the root diverge (AAP, 1992). Furcation involvement (FI) refers to the condition when periodontal disease has caused bone resorption into the bifurcation or trifurcation of a multirooted tooth [1]. The classification of furcation bone loss is based on both horizontal and vertical classifications. The horizontal classifications include Goldman (1958), Hamp (1975), and Ricchettie (1982), while the vertical classifications include Glickman (1953) and Tarnow (1984) [2]. Tarnow's classification of teeth with furcation involvements refer mainly to the vertical component of bone loss. Based on the vertical component, each grade of furcation is divided into three subgroups depending on the distance between the bottom of the defect and the roof of the furcation. The subclasses are A (1-3 mm), B (4-6 mm), and C (>7 mm) [3].

The involvement of the molar furcation area is one of the most common consequences of periodontitis; in fact, several retrospective studies have suggested that molars with furcation involvement have a compromised prognosis and respond less favorably to periodontal therapy. Detection of these defects primarily depends on clinical examination, particularly in mild and moderate periodontal bone loss. However, in severe periodontal bone loss, panoramic and intraoral radiographs are very important diagnostic tools, along with clinical examination [4].

Cone beam computed tomography (CBCT) was established in 1996 in the European market and approved by FDA in the United States in 2001. CBCT uses a cone-shaped X-ray beam and area detector that acquires a full volume of images in single rotation, and patient movement is not required. The software allows reconstruction by obtaining axial coronal and sagittal planes. By examining the 3D volumes generated by CBCT scans, addition anatomic information about the furcation lesions can potentially be obtained [5].

The advantages of using CBCT include evaluation of all possible sites and anatomical structures, no superimposition, the magnification is uniform. Additionally, the cost and the effective dose is less than that of multidetector CT (MDCT). In fact, the effective dose of CBCT is around 45 times lower than MDCT [6].

CBCT imaging has been applied in oral and maxillofacial surgery and is still the most common referral for CBCT. Surgical indications for CBCT include evaluation of impactions and the extent of the lesions in the jaws, as well as implant site assessment. In addition, CBCT is prescribed to visualize pre- and postsurgical sites. CBCT when used for evaluation of airway space of patients with sleep apnea may be useful in deciding the surgical procedure [7].

CBCT limited FOV is used in endodontics to detect vertical root fractures; it is useful for detection of supernumerary canals and complex anatomy, identification of calcified canals, and visualization of nonresolving periapical lesions. CBCT scans are used for evaluation of endodontics complications, such as overextended root canal materials, perforations, and visualization of separated endodontic instruments. Justification for prescribing CBCT should be consistent with ALADA (as low as diagnostically acceptable) principles [8].

The prognosis and periodontal treatment of the tooth depends on several key factors such as diagnosis and accurate determination of the location and the extension of the bone defects and also the furcation defect classification.

Presently, clinical probing and intraoral radiograph are the main tools for diagnosis of the diseases of the periodontium, but both the tools have limited application in assessing the periodontal bone loss. The major drawback being the inability to achieve 3D data of the periodontal bone defects such as furcation and intrabony defects. Therefore, in order to understand the bone dimensions and to evaluate the bone gain after treatment, direct surgical or open bone measurements was considered to be the gold standard. But, even with this procedure, limited time was available for the surgeon during the surgery to access the type and depth of the periodontal defect and to plan for periodontal regenerative treatment. Therefore, CBCT which allows for 3D evaluation of the dentition along with the surrounding supporting structures was introduced to deal with such limitations [9].

Recent studies have shown that combination of computed microtomography (*μ*CT) and magnetic resonance imaging (*μ*MRI) improve the quality of information obtained about bone tissue heterogeneity and can allow the researchers and clinicians to nondestructively characterize and follow up bone regeneration [10].

The limitations for the use of CBCT for diagnosis and treatment planning in the management of periodontitis, at this time, increased evidence for CBCT imaging in detecting interfurcal, vertical, and horizontal bone loss. With respect to the teeth and sites analyzed, the benefit of CBCT imaging varies and is particularly pronounced in maxillary molars [11, 12]. Further research is needed to determine the utility of CBCT imaging in supporting minimally invasive therapies, in assessing periodontal regenerative outcomes, and in determining the necessity of combination therapy (orthodontics, guided periodontal tissue regeneration, and soft tissue grafting) in complex cases. In addition, the development of new cost-effective approaches to CBCT imaging is also indicated [13].

Periodontal probing is the clinical method of choice for measuring furcation bone loss. In some cases, such as tooth position, and clinician technique, morphologic variations such as cervical enamel projections, presence of root concavities and enamel pearls, and presence of inflammation may affect the accuracy of measurements [14]. Two-dimension radiographs (bitewings and panoramic) are routinely used as adjunctive with clinical examinations. A furcation arrow has been used as a clinical indicator for the furcation defects. However, the literature suggests that this indicator has limited usefulness in terms of detection of furcation invasion [15]. Visualization of furcation defects in intraoral radiographs has many limitations, such as overlapping of anatomic structures, distortion, and difficulty in distinction between buccal and lingual cortical plate due to lack of 3D information [16, 17]. Therefore, additional imaging is required to improve the diagnostics information for furcation defects.

Hence, the aim of the study was to compare the measurements of trifurcation bone loss, measured using CBCT, versus clinical measurements in order to evaluate the efficacy of CBCT as an adjunctive diagnostic tool.

## 2. Material and Methods

Based on a previous study which compared periodontal defects including furcation assessment between CBCT and clinical probing, a power analysis was performed which demonstrated that a sample size of 38.06 subjects would achieve 80% power, which was rounded to 40 [9]. Therefore, the study group comprised of 40 patients (25 males and 15 females) with an average age of 56.6 years.

The Rutgers biomedical and health sciences (RBHS) IRB has approved this study. Clinical periodontal measurements were performed by a periodontics resident and CBCT measurements obtained by an Oral Radiology resident. Patients with furcation defects in maxillary molars include A, B, and C Tarnow classification. Included patients had both CBCT scans for maxillary molars and completed periodontal charts. Mandibular scans and incomplete periodontal charts were excluded from the study.

Clinical examination consisted of probing and detection of vertical and horizontal furcation defects. These were measured and recorded. CBCT measurements were then evaluated using the linear measuring tool in Carestream imaging software (Carestream, Rochester, USA) and iCAT (Imaging Sciences, Hatfield, USA). These measurements of the CBCT images were then documented and compared to clinical findings. The two examiners were blinded to each other's measurements.

### 2.1. Inclusion Criteria

Patients with periodontal bone loss in maxillary posterior area and patients for whom CBCT scans were indicated for the maxillary posterior area were included.

### 2.2. Exclusion Criteria

Patients under 18 years old, any systemic disease affects quantity or quality of alveolar bone, patients CBCT mandibular only scans, and maxillary scans with missing posterior teeth were not included.

### 2.3. Clinical Measurements for the Defects

Initial therapy including oral hygiene instructions, scaling and root planing using ultrasonic devices, and hand instruments was carried out. After which, the clinical bone measurement was made and recorded by a periodontics resident using Tarnow's classification of (vertical) furcation measurements. The measurements were made using a periodontal probe graded in millimeters (PCPUNC-15: HU-Friedy, Chicago, IL, USA). The measurement was taken from furcation entrance to the level of the resorbed bone.

### 2.4. Cone Beam Computed Tomography CBCT Measurements

The measurements were made by two calibrated Oral and Maxillofacial Radiology residents of the maxillary first and second molars at three locations: buccal furcation, mesial furcation, and distal furcation. All CBCT scans prescribed to the patients for endodontic and periodontic purposes used limited field of view. The CBCT scans were exposed for prior indication, and no patient had a scan advised solely for the purposes of this study. The measurements included the area from the entrance of the furcation to base of the defect. Linear measurements were performed by Invivo software (Anatomage, San Jose, USA). The scans were viewed and analyzed in coronal, sagittal, and cross-sectional planes. All measurements were recorded using the measurement tools included in the software.

### 2.5. Statistical Analysis

Paired *t*-tests were used to assess means of measurements for CBCT and clinical measurements. A general linear model was used to compare the outcome of the mean of the difference of the 2 systems CBCT and the clinical parameter between the levels of each independent variable. These were the classifications 1 and 2 as well as age and gender.

## 3. Results

### 3.1. Demographic Data

The number of males were 25 (63.4%) and the females 15 (26.5%). The mean value for the age is 56.6.

The most common tooth with a detected furcation defect was tooth #2 (31.7%), followed by tooth #15 (26.8%) and #3 (21.9%). The least common tooth with a detected furcation defect was #14 (19.5%).

**Figure 1:**
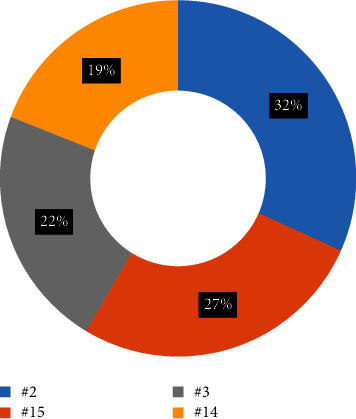


**Figure 2:**
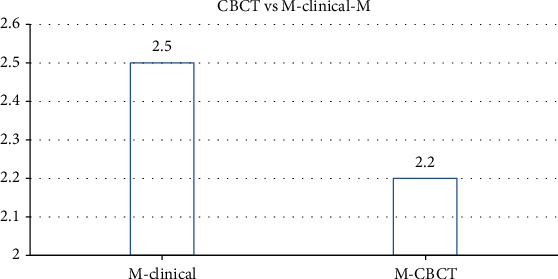


**Figure 3:**
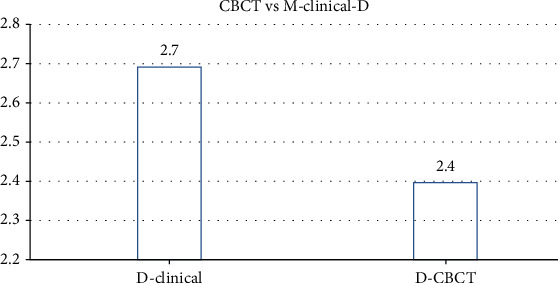


**Figure 4:**
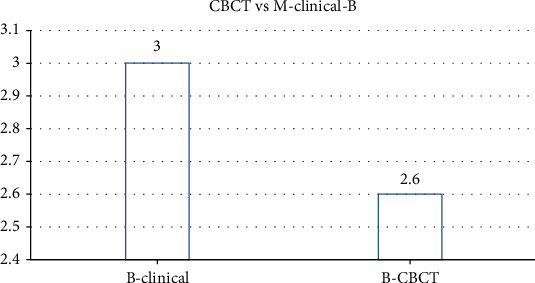


The prevalence of vertical furcation defects in this study are shown in Figure 1. The measurements of mesial furcation clinically were 2.5 mm and 2.2 mm for CBCT (Figure 2). The distal measurement of clinical examination was 2.7 mm and 2.44 mm for CBCT (Figure 3). The mean values of buccal furcation for clinical and CBCT measurements were 3.01 mm and 2.6 mm, respectively (Figure 4).

### 3.2. Data Analysis

Paired sample *t*-tests were used to look at mean value differences between CBCT and clinical measurements for the buccal, mesial, and distal sites. There was significant difference between the CBCT and clinical measurements for the buccal site (*p* = 0.010), and almost significant for the mesial site (p = 0.05), but not for the distal sites (*p* = 0.187) as seen in Table 1.

A general linear model (GLM) was used to compare outcomes of the mean of the difference of the 2 systems CBCT and clinical, between the levels of each independent variable which were classification 1 (Tarnow subclass A), classification 2 (Tarnow subclass B), gender, and age, as seen in Table 2. The dependent variables or outcomes are the differences of clinic and CBCT measurements in buccal, mesial, and distal sites. The absolute values of differences were used for the data analysis.

**Figure 5:**
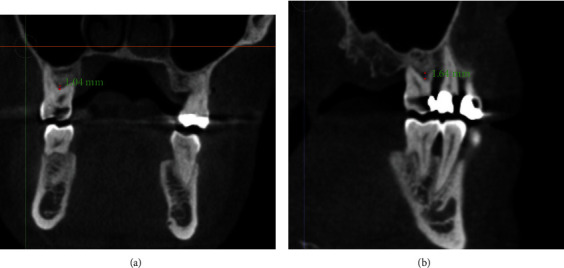


**Figure 6:**
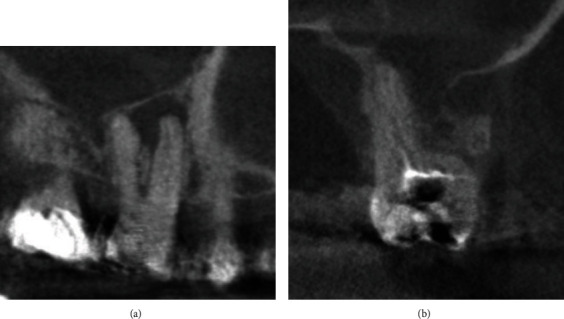


Figure 5 indicates coronal and sagittal scans showing furcation defects taken by ICAT (Imaging Sciences, Hatfield, USA), whereas Figure 6 indicates the coronal and sagittal scans showing furcation defects taken by Carestream, Rochester, USA.

Classification 1 was statistically significant for the difference of two system measurements in the buccal (*p* = 0.017), mesial (*p* = 0.005), and distal (*p* = 0.003) areas. The classification 1 with less than 3.5 mm had significant higher mean difference than the one with large or equal to 3.5 mm in all 3 sites.

## 4. Discussion

The purpose of this research was to evaluate the difference between CBCT linear measurements and clinical measurements in trifurcation defects in maxillary molar teeth. From our results, we found that there was no significant difference between CBCT and clinical probing in mesial and distal furcation. However the buccal furcation was found to be statistically significant. Although significant, a difference of 0.3 mm is not clinically meaningful or relevant for patient outcomes.

An animal study compared periapical radiographs and CBCT in mandibular pig's teeth at the bifurcation area. The study concluded that CBCT had higher sensitivity and specificity than periapical radiograph in simulating bony defects in buccal and lingual aspects of the mandible. This study is only applicable to mandibular teeth that include two-rooted teeth and not relevant to the maxillary teeth, due to presence of palatal root which can get superimposed on the adjacent bony structures. In our study, we included only maxillary molars (three-rooted) which could be difficult to visualize using 2D imaging [18].

Another study conducted in Germany prepared periodontal defects in vitro. All images were visualized by 15 dentists. The results with regard to furcation defects were similar to our result, which indicated that CBCT is better than two-dimensional radiographs to detect class II lesions. However, in our study, we compared CBCT to clinical measurements [19].

A comparative study conducted by Bagis et al. concluded that CBCT has highest sensitivity in terms of diagnostic accuracy between two examiners. The study was performed in 12 dry skulls to evaluate periodontal defects. Since the study was done on dry skulls, the measurements could be altered if the measurements were taken on the patients due the tongue movement and presence of the cheeks. Kappa values in this study were 0.78 and 0.96 for CBCT compared to 0.43 and 0.73 for intraoral radiographs. The present study uses human subjects, whereas Bagis et al. included dry skulls with wax to simulate soft tissue [20].

A study done in Basel, Switzerland, found that 84% of the CBCT measurements were confirmed by intrasurgical measurements, with Kappa score 0.926 indicating a high level of accuracy. In the present study, probing measurements rather than intrasurgical measurements were compared. The mean value difference in the present study was 0.5 mm between CBCT and clinical measurements for buccal furcation defects. For mesial and distal defects, the mean values were 0.3 mm and 0.24 mm, respectively. In addition, Walter et al. found 44% were underestimation for evaluating maxillary molars. Results of the present study exhibits that the mean value of CBCT measurements underestimates the clinical measurements in all sites [21, 22].

A similar study was conducted on patients with generalized chronic periodontitis after initial therapy. CBCT was conducted in upper molars with 6 mm of probing depths and advanced furcation involvement after which CBCT images were examined. It was concluded that CBCT images were more accurate in assessing the loss of periodontal tissue of the furcation involvement and root morphologies in upper molars [23]. In another comparative study between periodontal probing and CBCT, the number of FI detected by means of CBCT was larger than by means of periodontal probing [24].

Walter et al. studied the financial benefits of using CBCT for the management of maxillary molars furcations. The results from this study clearly state that prescribing CBCT leads to a reduction in treatment cost and time. This may be particularly true in invasive periodontal procedures due to a potential reduction in complications [25].

According to international commission on radiological protection (ICRP) 2007 guideline, the radiation dose is expressed as effective dose. Effective dose, measured in Sieverst (Sv), is more relevant than absorbed dose to measure the predicted stochastic effects. Effective dose measures the type, sensitivity, and likelihood of carcinogenesis of the irradiated tissues [26–28].

Since CBCT imaging uses a limited field of view (FOV), it offers greater benefits during the evaluation and management of furcation defects. Along with this advantage for the clinician, it exposes the patient to a minimal dose of ionizing radiation. Our study does not rule out the need for the clinical periodontal examination, but it can justify the use of CBCT scans by the clinicians, especially the periodontist, to evaluate furcation defects in maxillary molars using a small field of view.

### 4.1. Strengths and Limitations

As mentioned in the literature review, most of the studies were done on dry skull and mandible of pigs. However, the present study was done on human subjects. Most of the previous studies evaluated horizontal component of furcation defects, whereas our study focuses on the vertical component of furcation defects as limited data is available on it.

However, the main limitation of the study other than its cost was that it was an observational study and such studies are subjected to bias. The difference in the measurements bias between examiners can impact the internal validity. Lack of randomization is an added drawback of the study. Lastly, the current study only measured vertical bone loss and Tarnow's classification was used in the clinical detection. Future studies could consider incorporating both horizontal and vertical bone loss measurements on CBCT.

## 5. Conclusion

CBCT can be used as an adjunct to clinical furcation measurements and adds useful diagnostic information to assess trifurcation defects. In addition, CBCT limited field of view (FOV) can provide relatively high resolution images at a reduced dose that is comparable to two-dimensional imaging.

## Figures and Tables

**Table 1:** Descriptive statistics and the result of paired sample *t*-tests.

	*N*	Mean difference	Std. deviation of difference	*p* value
Buccal 1 buccal2	41	0.29	0.67	0.010^∗^
Mesial 1 mesial2	41	0.29	0.92	0.050
Distal 1 distal 2	41	0.24	1.16	0.187

**Table 2:** Descriptive statistics for differences of two system measurements and a result of GLM (^∗^statistically significant at *p* < 0.05. Note: full GLM was performed. For all two-way and three-way interactions, *p* values > 0.05).

			*N*	Mean	Standard deviation	*p* value
Difference in buccal	Classification 1	<3.5 mm	20	0.6	0.7	0.017^∗^
		≥.5 mm	21	0.4	0.2	
	Classification 2	<4 mm	21	0.5	0.6	0.089
		4-7 mm	20	0.5	0.4	
	Gender	Male	26	0.4	0.4	0.054
		Female	15	0.6	0.7	
	Age					0.787
Difference in mesial	Classification 1	<3.5 mm	20	1.0	0.7	0.005∗
		≥3.5 mm	21	0.5	0.4	
	Classification 2	<4 mm	21	0.8	0.7	0.191
		4-7 mm	20	0.6	0.6	
	Gender	Male	26	0.6	0.5	0.069
		Female	15	0.9	0.8	
	Age					0.734
Difference in distal	Classification1	<3.5 mm	20	1.2	0.7	0.003^∗^
		≥3.5 mm	21	0.7	0.6	
	Classification2	<4 mm	21	1.0	0.7	0.115
		4-7 mm	20	0.9	0.7	
	Gender	Male	26	0.8	0.6	0.011^∗^
		Female	15	1.2	0.8	
	Age					0.242

## Data Availability

The data that support the findings of this study are available from the corresponding author, upon reasonable request.

## Abbreviations

AAP: American Academy of Periodontology
CBCT: Cone beam computed tomography
FI: Furcation involvement
FOV: Field of views
GLM: General linear model
ICRP: International commission on radiological protection
Sv: Sieverst.
